# Exogenous Melatonin Alleviates Cold Stress by Promoting Antioxidant Defense and Redox Homeostasis in *Camellia sinensis* L.

**DOI:** 10.3390/molecules23010165

**Published:** 2018-01-15

**Authors:** Xin Li, Ji-Peng Wei, Eric R. Scott, Jian-Wei Liu, Shuai Guo, Yang Li, Lan Zhang, Wen-Yan Han

**Affiliations:** 1 Key Laboratory of Tea Quality and Safety Control, Ministry of Agriculture, Tea Research Institute, Chinese Academy of Agricultural Sciences, 9 Meiling Road, Hangzhou 310008, China; lixin@tricaas.com (X.L.); jipengwei93@163.com (J.-P.W.); liyang902102@163.com (Y.L.); zhanglan@tricaas.com (L.Z.); 2 Department of Biology, Tufts University, Medford, MA 02155, USA; eric.scott@tufts.edu; 3 Agricultural Technology Extension Center of Fuyang District, 118 Guihua West Road, Hangzhou 330183, China; liujianwei269968@163.com; 4 Hangzhou Botanical Garden, 1 Taoyuanling, Hangzhou 310013, China; changeyihao2008@126.com

**Keywords:** *Camellia sinensis*, tea plant, melatonin, cold stress, antioxidant enzymes, redox homeostasis

## Abstract

The unprecedented early spring frost that appears as a cold stress adversely affects growth and productivity in tea (*Camellia sinensis* L.); therefore, it is indispensable to develop approaches to improve the cold tolerance of tea. Here, we investigated the effect of pretreatment with exogenous melatonin on the net photosynthetic rate, the maximum photochemical efficiency of PSII, chlorophyll content, lipid peroxidation, reactive oxygen species (ROS) accumulation, antioxidant potential, and redox homeostasis in leaves of tea plants following cold stress. Our results revealed that cold treatment induced oxidative stress by increasing ROS accumulation, which in turn affected the photosynthetic process in tea leaves. However, treatment with melatonin mitigated cold-induced reductions in photosynthetic capacity by reducing oxidative stress through enhanced antioxidant potential and redox homeostasis. This study provides strong evidence that melatonin could alleviate cold-induced adverse effects in tea plants.

## 1. Introduction

Tea is considered as the most popular non-alcoholic beverage throughout the world. Green tea is manufactured from young leaves and buds of *Camellia sinensis* L. It is one of the most essential economic crops in some regions of the world [1,2,3]. Although this evergreen perennial plant can be grown in various agro-climatically diverse regions [4,5], the majority of tea production is limited to tropical and subtropical climates due to its thermophilic nature [4]. Every year, the fresh leaves and buds are plucked from the very early spring growth for tea manufacturing. Because of the poor tolerance of young leaves and buds to low temperature, usually a sudden frost in early spring causes cold stress and adversely affects the commercial yield of tea [6,7,8,9]. The combination of warmer maximum temperatures leading to earlier budburst and increased temperature variance under climate change is expected to lead to greater risk of early spring frost damage [10]. Therefore, it is indispensable to better understand the physiological response to cold stress and explore ways to improve cold tolerance in tea plants [11,12].

In plants, cold stress exerts various effects, leading to changes in physiology, biochemistry, and molecular biology [13,14,15]. Cold stress directly affects the photosynthetic machinery, principally by inducing photoinhibition at both photosystem I (PSI) and PSII [16]. Furthermore, the defects in the electron transport chain in chloroplasts cause excessive generation of reactive oxygen species (ROS) [17,18]. Over accumulation of ROS induces an increase in membrane lipid peroxidation, as well as damage to vital biomolecules [19]. Therefore, plants have developed a well-equipped antioxidant defense machinery that comprises both enzymatic and non-enzymatic antioxidants to protect cells against detrimental effects of ROS [17,19]. Cold stress-induced diminution in the electron flux for photosynthetic carbon reduction is closely associated with a significant enhancement in the activity of enzymatic antioxidants including superoxide dismutase (SOD) and ascorbate peroxidase (APX) in leaves [20]. Under cold stress, glutathione, a vital non-enzymatic antioxidant, also plays a critical role in redox homeostasis and defense-related signaling in plants [21]. Because the molecular mechanisms of plant tolerance to cold are reasonably well understood, recent studies are largely focused on the strategies towards improvement of the plant resistance against cold stress [10,22].

Melatonin (*N*-acetyl-5-methoxytryptamine) is an evolutionarily conserved molecule found in almost all living organisms including plants [23]. For a long time, our understanding of melatonin was limited to animals. However, it has been well documented in plants during recent years, since it was first reported in plants in 1995 [24,25,26]. In plants, melatonin occurs in all organs and acts as an essential signaling molecule that safeguards against environmental stresses including heat and cold [27,28,29,30,31,32]. Moreover, melatonin is principally considered as a critical antioxidant in plants that rapidly scavenges ROS when plants encounter a variety of stresses [33]. Melatonin also regulates activities of antioxidant enzymes and redox status through transcriptional upregulation of respective genes, especially under biotic and abiotic stress [32].

Although occurrence of melatonin in Chinese green tea and black tea has been confirmed [34], there has been no further research to explore the functions of melatonin in tea plants. Recently, we showed that cold stress-induced alterations in biochemical and physiological processes eventually affect the growth and quality of tea [4,6]. However, a few studies have been conducted exploring physiological strategies that improve cold tolerance in tea plants [10,22]. In this study, we attempt to investigate the effect of pretreatment with exogenous melatonin on the net photosynthetic rate, maximum photochemical efficiency of PSII, chlorophyll content, lipid peroxidation, antioxidant potential, and redox homeostasis in tea leaves under cold stress. Our results advocate that melatonin can enhance tea plants tolerance to cold stress through improving antioxidant defense and redox homeostasis.

## 2. Results

### 2.1. Melatonin Improves Photosynthetic Rate in Tea Plants under Cold Stress

**Figure 1:**
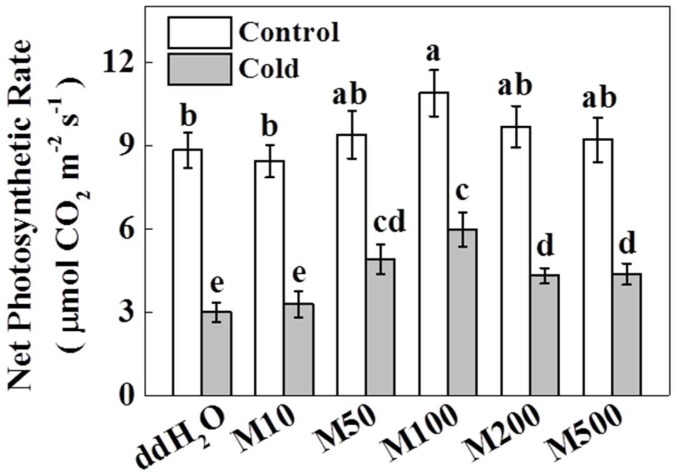
Net photosynthetic rate (Pn) as influenced by various concentrations of exogenous melatonin following cold stress. Two years-old tea (*Camellia sinensis* L.) seedlings of Longjing 43 were pretreated with various concentrations of melatonin (10, 50, 100, 200, and 500 µM) and challenged with cold stress (−5 °C for 3 h) in temperature-controlled chambers. Pn was measured in mature tea leaves after a 3 h recovery from cold stress. Each data point presents the mean of six replicates (± S.D. (standard deviation)). Means denoted by different letters are significantly different (*p* < 0.05) based on the Tukey’s test.

Plant response to melatonin is largely dose dependent [29,35]. To elucidate the effect of melatonin on photosynthesis in tea plants under cold stress, we pretreated foliage of tea plants with a series of melatonin concentrations between 10 µM and 500 µM. Melatonin was applied three times with applications every five days. Twenty-four hours after the final application of melatonin, the plants were either challenged with cold stress (−5 °C for 3 h) or kept in normal temperature conditions. After a recovery period in 25 °C conditions, net photosynthetic rate (Pn) was measured in tea leaves. In the absence of cold stress, 100 µM melatonin caused a significant increase (23.3%) in Pn, while other concentrations of melatonin had no effect on Pn (all *p* < 0.05). Furthermore, as shown in Figure 1, Pn decreased by 66.2% after cold stress without melatonin pretreatment. Though the lowest concentration of melatonin (10 µM) had no effect on Pn, all other melatonin concentrations caused a significant increase in Pn after cold stress, with the greatest increase in Pn value at 100 µM melatonin (99.5% greater than that in ddH_2_O). Higher concentrations of melatonin (200 µM and 500 µM) did not increase Pn as much, but were still significantly greater than ddH_2_O (46.1% greater at 500 µM). These results suggest that, except for 10 µM, all the concentrations of melatonin positively stimulate photosynthesis in cold-treated tea plants. Both in the presence and absence of cold stress, melatonin promoted the Pn in a concentration dependent manner. Based on the above results, we chose 100 µM melatonin as the best concentration for the rest of our measurements.

### 2.2. Effect of Melatonin on Chlorophyll a Fluorescence in Tea Leaves under Cold Stress

**Figure 2:**
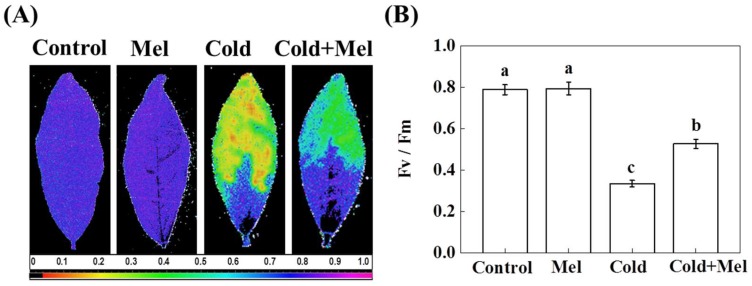
Effect of melatonin application on the maximum photochemical efficiency of PSII (Fv/Fm) in tea leaves under normal and cold stress conditions. (**A**) Pseudo color image of Fv/Fm; and (**B**) Fv/Fm value under different treatments. Pseudo color gradient shown at the bottom of the image ranges from 0 (black) to 1 (purple). Mel, melatonin. Each data point presents the mean of six replicates (± S.D. (standard deviation)). Means denoted by different letters are significantly different (*p* < 0.05) based on the Tukey’s test.

We measured chlorophyll a fluorescence as a proxy for stress-induced damage to the photosynthetic apparatus. Notably, Fv/Fm is an essential chlorophyll a fluorescence parameter that is sensitive to cold stress [36]. Fv/Fm can be qualitatively observed by pseudo-color images, which indicate status of the photosystem II [37]. In Fv/Fm images, purple-blue color indicates normal state of photosynthetic apparatus, while green and yellow colors indicate damage to photosystem II caused by cold (Figure 2A). Quantitative analysis of Fv/Fm values revealed that exposure to cold stress significantly decreased Fv/Fm by 57.6% (Figure 2B). Though melatonin application has no effect on Fv/Fm without cold stress, melatonin pretreatment caused a significant increase in Fv/Fm (57%) under cold stress.

### 2.3. Effect of Exogenous Melatonin on Malondialdehyde (MDA), Hydrogen Peroxide (H_2_O_2_), Chlorophyll Content, and Superoxide (O_2_^●−^) Accumulation in Tea Leaves under Cold Stress

**Figure 3:**
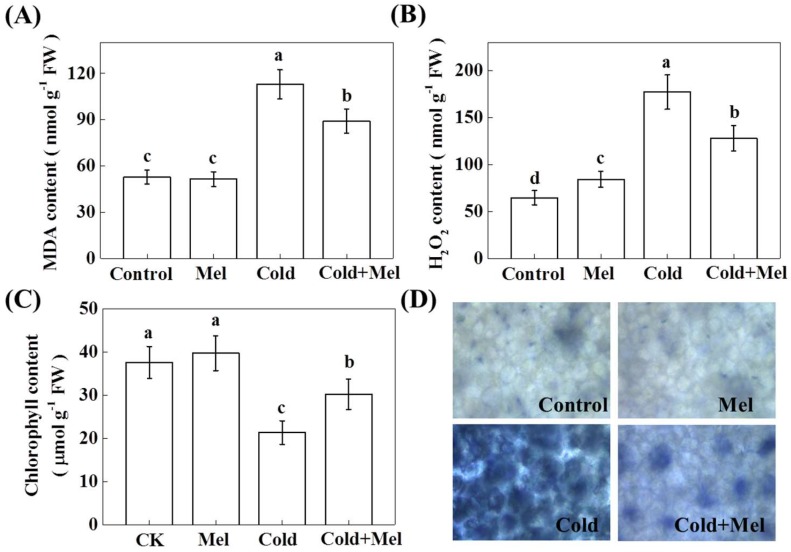
Effect of melatonin application on different stress-related parameters. (**A**) malondialdehyde (MDA) content; (**B**) hydrogen peroxide (H_2_O_2_) content; (**C**) chlorophyll content and (**D**) in situ O_2_^●−^ accumulation by NBT staining. Mel, melatonin. Each data point presents the mean of six replicates (± S.D. (standard deviation)). Means denoted by different letters are significantly different (*p* < 0.05) based on the Tukey’s test.

Environmental stress generally triggers ROS production, leading to membrane lipid peroxidation [17]. To examine whether exogenous melatonin could protect plants from cold-induced lipid peroxidation, we analyzed the relative levels of MDA and H_2_O_2_. Cold stress significantly increased MDA level by 114.2% in tea samples after cold stress treatment (Figure 3), suggesting that cold treatment caused severe oxidative stress in tea plants. In contrast, tea seedlings that were pretreated with exogenous melatonin accumulated much less MDA (78.9%, compared with that of cold stress treatment) when they were later subjected to cold stress, while exogenous melatonin had no effects on MDA concentration without cold stress.

Analysis of H_2_O_2_ content showed that cold stress significantly increased H_2_O_2_ level by 273.9% compared with that of non-stressed control tea plants (Figure 3). In contrast, exogenous melatonin application before the cold stress significantly inhibited the increase of H_2_O_2_ content by 27.8%, compared with that of cold stress treatment. Although no stress sign was observed in only melatonin treated plants, melatonin-only treatment induced an increase of H_2_O_2_ by 30.2%, indicating a potential signaling role of H_2_O_2_ in melatonin-mediated response. Quantification of total chlorophyll content in tea leaves showed that cold stress decreased chlorophyll content by 43.2%. Although exogenous melatonin had no effect on the chlorophyll content under normal temperature conditions, melatonin application on cold-treated plants significantly increased total chlorophyll content. NBT staining further confirmed an increased accumulation of O_2_^●−^ following exposure of plants to cold stress; however, exogenous melatonin treatment reduced cold-induced ROS accumulation when compared with that of only cold stress treatment (Figure 3D). 

### 2.4. Activity of Antioxidant Enzymes as Influenced by Exogenous Melatonin and Cold Stress in Tea Plants

**Figure 4:**
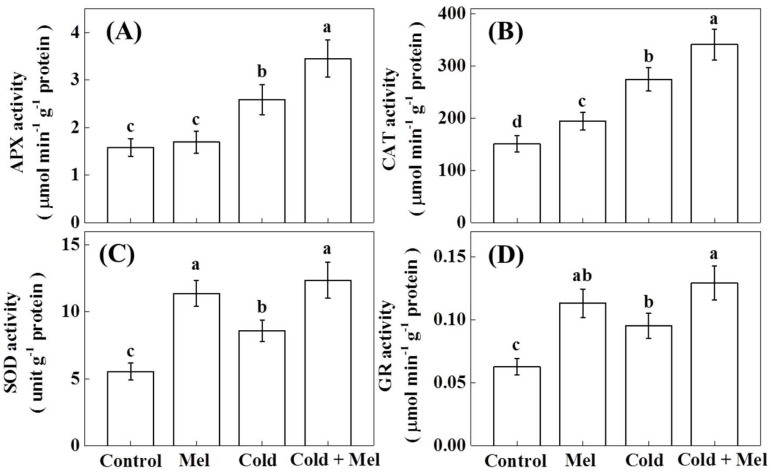
Effect of melatonin application on (**A**) ascorbate peroxide (APX), (**B**) superoxide dismutase (SOD), (**C**) catalase (CAT), and (**D**) glutathione reductase (GR) activities in tea leaves in the presence or absence of cold stress treatment. Mel, melatonin. Each data point presents the mean of six replicates (± S.D. (standard deviation)). Means denoted by different letters are significantly different (*p* < 0.05) based on the Tukey’s test.

Antioxidant enzymes play a pivotal role in protecting plants from oxidative stress. We found that activities of SOD, APX, catalase (CAT), and glutathione reductase (GR) significantly increased under cold stress (Figure 4). Enzyme activity was most strongly upregulated by cold stress for CAT (81.6%), followed by APX (63.2%), SOD (55.1%), and GR (51.7%) in tea seedlings. Melatonin application alone (without cold stress) increased enzyme activity also. Melatonin application increased enzyme activity of SOD (105.6%), followed by CAT (81.6%), and GR (80.2%). After cold stress treatment, the highest activity increase due to melatonin pretreatment was also for SOD (44.1%) and GR (35.6%). Interestingly, in most cases, exogenous melatonin resulted in an additional increase in the activity of those enzymes compared with that of cold stress alone.

**Figure 5:**
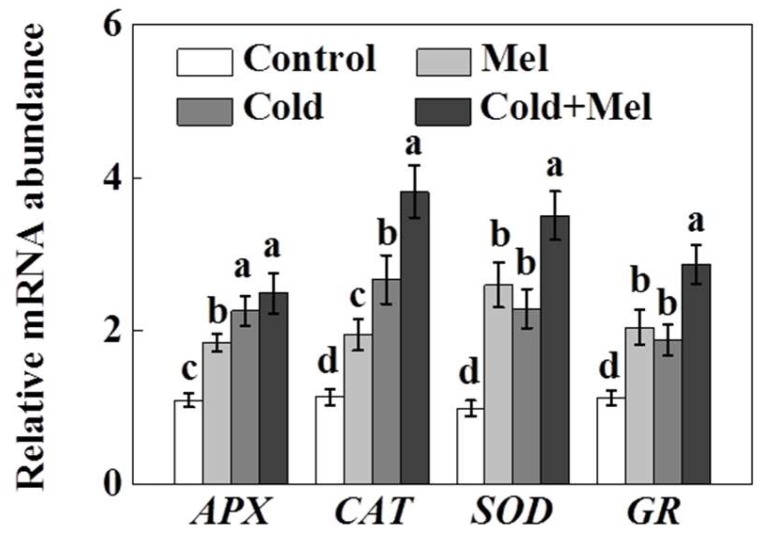
Effect of melatonin application on the transcripts of genes for ascorbate peroxide (APX), superoxide dismutase (SOD), catalase (CAT), and glutathione reductase (GR) in tea leaves in the presence or absence of cold stress treatment. Mel, melatonin. Each data point presents the mean of six replicates (± S.D. (standard deviation)). Means denoted by different letters are significantly different (*p* < 0.05) based on the Tukey’s test.

Similar to the enzyme activity, the expression of genes encoding different antioxidant enzymes, such as APX, CAT, SOD, and GR, significantly up-regulated following melatonin application or cold stress (Figure 5). Furthermore, melatonin pretreatment before cold stress significantly up-regulated the transcript levels of those genes (except for APX) compared with that in cold stress alone.

### 2.5. Effect of Melatonin on Redox Homeostasis of Tea Plant under Cold Stress

**Figure 6:**
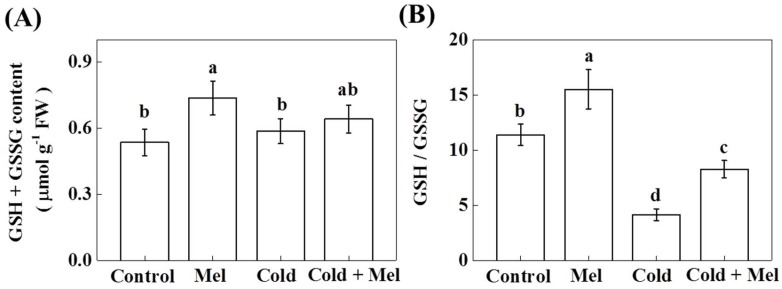
Effect of melatonin application on (**A**) glutathione content and (**B**) redox status in tea leaves under normal and cold stress conditions. Mel, melatonin. GSH, reduced glutathione. GSSG, oxidized glutathione. Each data point presents the mean of six replicates (± S.D. (standard deviation)). Means denoted by different letters are significantly different (*p* < 0.05) based on the Tukey’s test.

Except for antioxidant enzymes, nonenzymatic antioxidants, especially cellular glutathione, play essential roles in protecting plant cells against abiotic stress [21]. When reduced glutathione (GSH) scavenges ROS, it is oxidized to glutathione disulfide (GSSG). Later on, GSSG can be converted to GSH by GR. Therefore, we investigated the total glutathioine pool as well as the glutathione redox status measured as the ratio between reduced (GSH) and oxidized glutathione (GSSH) to better understand the mechanisms of melatonin-mediated enhanced tolerance to cold stress in tea plants. As shown in Figure 6, the glutathione pool (GSH + GSSG) in tea plants that faced cold stress remained unaltered. On the contrary, melatonin application before cold treatment significantly increased GSH + GSSG by 37.6%. Moreover, cold stress significantly decreased the ratio of GSH/GSSG by 63.5% in tea seedlings (Figure 6), whereas melatonin increased it at both normal and cold stress conditions, by 36.3% and 99.1%, respectively.

## 3. Discussion

Cold stress is one of the harshest abiotic stresses that tea plants experience. It negatively affects both yield and quality of tea [4,6]. Therefore, it is indispensable to develop approaches to improve cold tolerance of tea [7,9]. In the current study, we found that cold stress drastically decreased photosynthetic activity possibly by inducing excessive production of ROS. Furthermore, our data indicate that melatonin played an important role in ameliorating the negative effects of cold stress through up-regulation of antioxidant enzymes and cellular redox in tea plants. To our knowledge, this is the first report that explores the role of exogenous melatonin in enhancing plant tolerance to abiotic stress in tea plants.

Photosynthesis is a vital physiological process in plants, which is highly sensitive to changes in temperatures [38]. In the present study, cold stress significantly decreased Pn, as well as Fv/Fm. These results are in accord with previous reports that cold stress reduces Fv/Fm but promotes oxidative stress in both tea and tomato plants [6,39]. The diminution in Fv/Fm might be due to induced lipid peroxidation caused by excessive accumulation of ROS in tea leaves under cold stress [6,10]. This is consistent with previous research showing that excessive production of various ROS is the key reason behind the cold-induced photosynthetic inhibition [6,40]. Beside the role of ROS in pervasive damage, ROS cause oxidative degradation of the D1 protein [41,42] and damage to the oxygen-evolving complex subunits of PSII [43].

Our results revealed that certain concentrations of melatonin could promote photosynthesis in tea plants under cold stress. Likewise, Fv/Fm was significantly higher, while the levels of MDA, as well as H_2_O_2_, were significantly lower with melatonin pretreatment under cold stress. Those results suggest that an optimal level of melatonin could minimize the cold stress-induced damage to the photosynthetic machinery. Bajwa et al. [44] demonstrated that exogenous melatonin could enhance cold tolerance in *Arabidopsis thaliana*, which supports our current findings. Moreover, in our study, the effects of melatonin on photosynthesis under cold stress were concentration-dependent. Since endogenous melatonin concentrations vary from species to species, even among organs of the same plant [34], it is reasonable that different concentrations of melatonin exerted different effects on photosynthesis [45,46].

Analyses of enzyme activity and gene transcripts revealed that cold stress promoted APX, CAT, SOD, and GR, which is consistent with previous studies showing that CAT, SOD, and peroxidase (POD) activities are increased during cold stress [16,20]. Despite the increase in the activity of antioxidant enzymes, the levels of MDA and H_2_O_2_ increased significantly under cold stress, which further confirmed that cold treatment induced oxidative stress in tea plants. Similar enhancements in MDA and ROS production were observed in tomato following exposure to heat and salinity treatments, respectively [37,47]. Cold stress increased the concentration of MDA and H_2_O_2_, but this increase was significantly lower in plants exposed to cold stress after pretreatment with melatonin. This suggests that cold-induced oxidative stress was mitigated by melatonin pretreatment. Melatonin itself acts as a strong antioxidant and also triggers enzymatic antioxidants to minimize the rate of ROS production [48]. Turk et al. [49] reported that melatonin could induce the accumulation of antioxidant metabolism-related proteins to mitigate cold stress in wheat seedlings. Additionally, melatonin increases the potential of overall antioxidant systems to scavenge excessive free radicals [34,35,48]. For example, in *Malus* species melatonin not only scavenges H_2_O_2_, but also improves the activities of CAT and APX under drought stress [50]. Furthermore, under normal conditions, melatonin application increases CAT, SOD, and GR activity. We also observed that exogenous melatonin treatment induced accumulation of H_2_O_2_ in non-cold stressed tea plants. Our results are in agreement with previous reports that exogenous melatonin enhances tolerance to stress by inducing H_2_O_2_ as a defense signal in different plant species [33,51,52,53].

In addition to antioxidant enzymes, plants have to maintain an optimal cellular redox poise to continue normal metabolic activities [21]. As a redox active compound, glutathione maintains cellular homeostasis by influencing vital biological pathways, while abiotic stress-induced excessive accumulation of ROS disrupts redox poise [54]. Notably, GSH acts as a key antioxidant that directly decomposes H_2_O_2_ by converting itself into GSSG. GSH is further regenerated from GSSG by the activity of GR [21]. In the present study, cold stress significantly decreased the ratio of GSH to GSSG. Such changes in redox balance disrupt normal metabolic activity in tea plants. However, pretreatment with melatonin increased the GSH:GSSG ratio of cold-treated tea plants to a significantly high level (Figure 6), which was in accord with the effect of melatonin on redox homeostasis under cold stress in tomato [29].

To sum up, the present study showed that cold stress caused oxidative stress by triggering the production of ROS and MDA in tea leaves. Excessive production of ROS in turn inhibited photosynthesis by disrupting important cellular processes. On the contrary, treatment with melatonin improved the activity of antioxidant enzymes and redox homeostasis, leading to alleviation of cold-induced photosynthetic inhibition. Moreover, melatonin-mediated mitigation of oxidative stress was largely attributed to the upregulated transcript abundance of antioxidant genes. The conclusion is that the melatonin-induced tolerance of tea plants to cold stress provides new prospects for stabilizing tea production in tea growing regions at high risk of experiencing early spring frosts. To the best of our knowledge, this is the first report that unraveled the beneficial role of melatonin in cold tolerance in tea plants. However, further study is required to explore in-depth molecular mechanisms of melatonin-mediated cold tolerance in tea plants.

## 4. Methods

### 4.1. Plant Materials and Experimental Treatments

For the current study, two years-old tea seedlings (*Camellia sinensis* L.) were raised in a growth medium consisting of 6 parts peat, 3 parts vermiculite, and 1 part perlite (on volume basis) in plastic pots in controlled environment cabinets. Each pot contained one healthy seedling. “Longjing 43” cultivar was used, as it is the most popular cultivar for the production of green tea in China. Following growth conditions were maintained for raising seedlings: 600 µmol·m^−2^ s^−1^ photosynthetic photon flux density (PPFD), 12 h/12 h photoperiod (day/night), 25/20 °C air temperature (day/night), and 80% relative humidity. An optimum moisture level in the growth medium was uniformly maintained by daily watering, whereas Hoagland’s nutrient solution was used for fertilization with applications every 5 days. For dose trial, foliage of tea seedlings were sprayed with a set of melatonin (Sigma-Aldrich, St. Louis, MO, USA) concentrations (10, 50, 100, 200, and 500 µM). Melatonin was dissolved in 200 µL ethanol and then diluted with ddH_2_O to a final ratio of ethanol: water (*v*/*v*) = 1:10,000. To treat control plants, the same ratio of ethanol to ddH_2_O was used. Melatonin was applied three times with applications every five days. Twenty four hours after the last melatonin application, and tea seedlings were kept in −5 °C temperature for 3 h (cold stress) and then returned in 25 °C conditions to recover for 3 h. Tea plants that were not challenged with cold treatment served as controls. After the recovery period, net photosynthetic rate and maximum photochemical efficiency of PSII (Fv/Fm) were measured, and young leaves and buds were sampled for various biochemical and gene expression analyses. Six replicates were performed for each treatment, while one replicate comprised five seedlings.

### 4.2. Measurements of Net Photosynthetic Rate

Net photosynthetic rate was measured in the 2nd fully expanded tea leaves after a period of recovery from cold stress. Measurements were taken from 9:00 a.m. to 11:00 a.m. by an open-flow infrared gas analyzer adapted with light and temperature controlled systems (Li-COR 6400, Li-COR, Lincoln, NE, USA) maintaining 25 °C air temperature, 80% relative humidity, 400 µmol mol^−1^ CO_2_ concentration, and 600 µmolm^−2^·s^−1^ PPFD [6].

### 4.3. Determination of Fv/Fm

Prior to measurement of chlorophyll fluorescence, tea plants were dark adapted for 30 min. Fv/Fm was measured in the 2nd fully expanded leaves by an imaging-PAM chlorophyll fluorimeter fitted with a computer-operated PAM-control unit (IMAG-MAXI; Heinz Walz, Effeltrich, Germany) as described previously [37].

### 4.4. Detection of Malondialdehyde (MDA), Chlorophyll, and Reactive Oxygen Species Accumulation in Tea Leaves

The level of lipid peroxidation was determined by measuring MDA equivalent using the 2-thiobarbituric acid (TBA) test. Briefly, 5 mL 0.1% (*w*/*v*) trichloroacetic acid (TCA) was added to 0.3 g tea leaf tissues for homogenization. The homogenates were collected in tubes and centrifuged at 10,000× *g* for 10 min. Afterward, 1 mL supernatant was collected and mixed with 4 mL 20% TCA with 0.65% (*w*/*v*) TBA. The mixtures were placed in a hot water bath and kept at 95 °C for 25 min. Then, the reaction was stopped by placing the mixtures in an ice bath. After cooling, the resulting mixtures were centrifuged at 3000× *g* for 10 min. Finally, the absorbance was recorded at 440, 532, and 600 nm. Calculation of the MDA equivalents was made according to Hodges et al. [55]. Leaf chlorophylls were extracted from the tea leaves in 80% acetone, and chlorophyll contents were analyzed colorimetrically [56] by a spectrophotometer.

For determination of H_2_O_2_, leaf samples (0.3 g) were ground in liquid nitrogen and homogenized in 3 mL of 1 M HClO_4_ at 4 °C. After centrifugation of the homogenates at 6000× *g* for 5 min, supernatants were collected. The pH of the supernatants was adjusted to 6.0 with 4 M KOH. Afterward, the supernatants were centrifuged again at 12,000× *g* for 5 min at 4 °C. The sample was then passed through an AG1 × 8 prepacked column (Bio-Rad, Hercules, CA, USA), and 4 mL double-distilled H_2_O was added to elute H_2_O_2_. To initiate reaction, resulting elute was mixed with 400 µL reaction buffer that contained 4 mM 2,2′-azino-di(3-ethylbenzthiazoline-6-sulfonic acid), 100 mM potassium acetate at pH 4.4, 400 µL deionized water, and 0.25 U of horseradish peroxidase. Absorbance was recorded at 412 nm to measure H_2_O_2_ content [57]. To visualize superoxide (O_2_^●−^) accumulation, tea leaves were incubated with *p*-nitro blue tetrazolium (NBT) solution (0.1 mg mL^−1^, pH 7.8) in dark [29]. NBT-stained tea leaves were observed and photographed with high magnification using a light microscopy system (Leica DM4000B & DFC425, Leica microsystem Ltd., Heerbrugg, Germany).

### 4.5. Assay of Antioxidant Enzyme Activity

Tea leaf materials (0.3 g) were homogenized with 3 mL of 25 mM HEPES buffer (pH 7.8) comprising 0.2 mM EDTA, 2 mM AsA, and 2% PVP. After centrifugation of the homogenates at 12,000× *g* for 20 min at 4 °C, the supernatants were collected. Antioxidant enzyme activity was determined using these supernatants. The protein content was determined according to the method of Bradford [58]. Ascorbate peroxide (APX) activity was determined following the Nakano and Asada [59] method. The method of Stewart and Bewley [60], which is based on the photochemical reduction of NBT, was used to assay superoxide dismutase (SOD) activity. A decline at 240 nm was recorded to measure catalase (CAT) activity according to the method of Patra et al. [61]. Glutathione reductase (GR) activity was assayed measuring the decline in the absorbance of NADPH at 340 nm following the method of Foyer and Halliwell [62].

### 4.6. RNA Isolation and Gene Expression Analysis

We used TRIzol reagent (Invitrogen, Carlsbad, CA, USA) to isolate total RNA from tea leaf samples. RNA samples were passed through a purifying column to remove genomic DNA. Superscript II (Invitrogen) was used for reverse transcription as instructed by the manufacturer. A list of the primers used for qRT-PCR assay can be found in Supplementary Table S1. We used a Step One Plus Real-Time PCR system (Applied Biosystems, Foster City, CA, USA) and Power SYBR Green PCR Master Mix (Applied Biosystems) for qRT-PCR analysis maintaining following conditions: denaturation at 95 °C for 3 min, followed by 40 cycles of denaturation at 95 °C for 30 s, annealing at 58 °C for 30 s, and extension at 72 °C for 30 s. Normalization of transcript abundance was accomplished compared to the polypyrimidine tract-binding protein (*PTB1*) gene, as descried by Hao et al. [63]. Relative gene expression was calculated according to the formulae developed by Livak and Schmittgen [64]. 

### 4.7. Determination of Glutathione Content

For the determination of glutathione content, 2 mL of 6% meta-phosphoric acid comprising 2 mM EDTA was added to 0.3 g tea leaf tissue for homogenization. The homogenates were then centrifuged at 12,000× *g* for 10 min at 4 °C, and the supernatants were collected for the determination of reduced glutathione (GSH) and oxidized glutathione (GSSG). The contents of GSH and GSSG were assayed following the method described by Rahman et al. [65]. In brief, the reaction mixture contained 0.2 mM NADPH, 100 mM phosphate buffer (pH 7.5), 5 mM EDTA, and 0.6 mM 5,5′-dithio-bis (2-nitrobenzoic acid). Next, we added 0.1 mL of the supernatant to the reaction mixture after neutralization with 0.5 M phosphate buffer (pH 7.5). The reaction was initiated through addition of 3 U of GR. The changes in absorbance at 412 nm were monitored for 1 min. To analyze GSSG content, 40 μL 2-vinylpyridine was added and kept at 25°C for 1 h to mask GSH. GSSG content was subtracted from total glutathione content (GSH + GSSG) to obtain GSH content.

### 4.8. Statistical Analysis

The experiment was conducted following a completely randomized design with six replications. All statistical analysis were conducted using software SAS 8.1 (SAS Institute Inc., Cary, NC, USA). Data were statistically analyzed using analysis of variance (AVONA) and the means were compared for significant (*p* < 0.05) treatment differences using Tukey’s test.

## Supplementary Materials

Supplemental Table S1: A list of the primer pairs used for the expression analysis of genes relating to antioxidant enzymes.
