# Supplementary material for: Exogenous Melatonin Alleviates Cold Stress by Promoting Antioxidant Defense and Redox Homeostasis in Camellia sinensis L
Source: Molecules. 2018 Jan 15;23(1):165. doi: 10.3390/molecules23010165 (PMC6017414; doi:10.3390/molecules23010165)
Supplement: Supplementary file 1 [file molecules-23-00165-s001.docx]

**Supplemental Table S1.** Primers used for real time RT-PCR assays.

| **Gene** | **Functional annotation** |  | **Primer pairs** |
| --- | --- | --- | --- |
| *PTB1* | *POLYPYRIMIDINE TRACT-BINDING PROTEIN* | GAAC01052498.1 | F: 5’-TGACCAAGCACACTCCACACTATCG-3’ |
|  |  |  | R: 5’-TGCCCCCTTATCATCATCCACAA-3’ |
| *APX* | *ASCORBATE PEROXIDE* | KR296654 | F: 5’-GCCTCATCGCTGAGAAGAAC-3’ |
|  |  |  | R: 5’-AAGTACCAGCAGAGTGCCAT-3’ |
| *CAT* | *CATALASE* | KR819178 | F: 5’-ATGAACGTGGTAGCCCTGAA-3’ |
|  |  |  | R: 5’-TGTGAGACTTTGGGTTGGGT-3’ |
| *SOD* | *SUPEROIXDE DISMUTASE* | AY694187 | F: 5’-GATGACGGAACTGCTTGCTT-3’ |
|  |  |  | R: 5’-ATCAGGGTCTGCATGGACAA-3’ |
| *GR* | *GLUTATHIONE REDUCTASE* | KF418080 | F: 5’-TGCACATGTGTGGAGAGGAT-3’ |
|  |  |  | R: 5’-ATACCCACTGTGGCATCCAA-3’ |
